# Contribution from the SAARC Region to Ophthalmic Research: A Bibliometric Analysis

**DOI:** 10.12669/pjms.41.2.10581

**Published:** 2025-02

**Authors:** Yousaf Jamal Mahsood, Rima Khan, Hira Wakil, Saima Farooq, Rashid Zia

**Affiliations:** 1 Dr. Yousaf Jamal Mahsood, MBBS, MHR, FICO, MRCSEd, FRCS, FCPS Department of Ophthalmology, Khyber Girls Medical College, Hayatabad Medical Complex, Peshawar, Pakistan; 2 Dr. Rima Khan, MBBS Department of Ophthalmology, Khyber Girls Medical College, Hayatabad Medical Complex, Peshawar, Pakistan; 3 Dr. Hira Wakil, MBBS Department of Ophthalmology, Khyber Girls Medical College, Hayatabad Medical Complex, Peshawar, Pakistan; 4 Dr. Saima Farooq, MBBS, FCPS Department of Ophthalmology, Khyber Girls Medical College, Hayatabad Medical Complex, Peshawar, Pakistan; 5 Dr. Rashid Zia, MBBS, MRCSEd East Kent Hospitals University NHS Foundation Trust, Ashford, GB, United Kingdom

**Keywords:** Authorship, Bibliometric analysis, Ophthalmology, Publications, Peer Review, Research priority, South Asia

## Abstract

**Objective:**

To review the contributions to the ophthalmic research from the SAARC region in the top twenty ophthalmology journals.

**Methods:**

This was a bibliometric study and the top 20 ophthalmology journals, as ranked by the Scimago Journal Rankings (SJR) from 2021 and 2022 (two years), were selected for analysis. Only original research articles were included in the analysis. Articles were analysed based on authorship, corresponding authorship, and research centres within the South Asian Association for Regional Cooperation (SAARC) region (Afghanistan, Bangladesh, Bhutan, India, Maldives, Nepal, Pakistan, and Sri Lanka). The main research topics were also examined. This data was recorded on Microsoft excel sheet and then descriptive analysis were calculated.

**Results:**

Of the total 4952 articles reviewed, 208 (4.2%) had authorship from the SAARC region. Of the 38079 authors in total, 1133 (2.97%) were from the SAARC region and 715 (63.1%) were male. The authors from India contributed the most (n=1074, 94.8%) articles. Of the 155 corresponding authors from SAARC, 150 (96.78%) were from India. Research centres from India were the leading contributors, (n=166, 93.26%). Out of all the publications, medical retina accounted for 1546 (31.22%) of the total articles.

**Conclusions:**

Compared to its share of the world’s population, the SAARC region’s research contribution to the top twenty ophthalmology journals is nominal.

## INTRODUCTION

Health research plays a vital role in defining a nation’s progress. The field of ophthalmology is constantly evolving to address complex eye health and vision impairment issues. Studies on the contribution of ophthalmic research have been conducted both globally as well as in Asia.^1^,^2^ Ophthalmology research is crucial in addressing eye health issues and improving clinical outcomes, particularly in the South Asian Association for Regional Cooperation (SAARC) region where a significant portion of the global population resides. SAARC accounted for 5.21% (US$ 4.47 trillion) of the world economy, 21% of the world’s population, and 3% of the world’s land area as of 2021.^3^ Previous studies have shown that countries from the SAARC region accounts for a small percentage of the world’s scientific output and lacks representation among the top-cited ophthalmology research.^4^ Despite making up 21% of the global population, just 4.14% of the world’s overall scientific output comes from South Asian nations.^5^

The SAARC region, founded in 1985, consists of eight member nations (Afghanistan, Bangladesh, Bhutan, India, Maldives, Nepal, Pakistan, and Sri Lanka). There have been research contributions from these countries in the field of ophthalmology, but it is unclear how much of that contribution has been published by top ophthalmology journals. There is a lack of in-depth understanding of the current state of ophthalmology research contribution from the SAARC region. Bibliometric analysis provides quantitative insights into the impact, trends, and quality of research, helping identify influential studies, authors, and emerging fields efficiently.^6^,^7^ This emphasizes the need for a comprehensive bibliometric analysis to characterize the state of ophthalmology research, identify dominant patterns, evaluate research publications, and pinpoint areas that require further improvement.

Since SAARC ophthalmologists’ publications in high-impact factor international journals have the potential to greatly impact the global landscape of ophthalmology research, therefore it is vital to explore publications in these journals. To the best of our knowledge, this is the first bibliometric analysis focusing on ophthalmology publications from the SAARC region in top ranked journals. Through a critical examination of the literature, this study aims to conduct a bibliometric analysis of top 20 ophthalmology journals and determine the contributions from the SAARC region. We hypothesize that the contributions of the SAARC region to top-tier ophthalmology journals are significantly less than its share of the world’s population and research output, and further research is needed to improve the field’s contribution to global ophthalmology research. This study will provide stakeholders, decision-makers, scholars, and medical professionals with information regarding the status of ophthalmology research in the SAARC area, hence stimulating efforts to improve eye health and vision care for the many populations it serves.

## METHODS

This study was conducted at the department of ophthalmology of Hayatabad Medical Complex, Peshawar. The 20 highest-ranking ophthalmology journals based on impact factor by SCImago (www.scimagojr.com) for the years 2021 and 2022 were selected.^8^,^9^ SCImago journal rankings (SJR) were preferred over other databases because it is an open access system. Only original research articles were included in this study to explore the research generation from primary source data from SAARC nations. Those articles which were retracted were excluded from our study. The information extracted from the selected articles included: the total number of authors, the number of authors from the SAARC region, gender-wise distribution of these authors, the number of research centres from SAARC countries, and the topic of research. All the data were recorded using an Excel spreadsheet. Data were collected by two independent investigators (RK and HW). Confusion and disagreement were resolved by a third investigator (YJM). The authors were stratified into SAARC countries based on their countries of affiliation. This was also applied to corresponding authors. To determine the gender of the authors accurately, a Google search was applied to find the authors’ profile page from their affiliated institution’s website. In cases of unavailability of this, a Google search was done to determine whether a name was associated with a male or female gender based on popular usage of a certain name on the internet as done in a previous study.^10^ The topics of research included: General Ophthalmology; Paediatric ophthalmology and Strabismus; Uveitis; Glaucoma; Cornea and External Diseases; Medical Retina; Vitreo-Retinal Surgery; Oculoplastic; Cataract Surgery; Ocular Oncology; Neuro-Ophthalmology; Ocular Pathology; Community Eye Health, and trauma. One topic of research was allocated to most articles. However, when varied subjects were discussed in an article, two topics of research (primary and secondary) were assigned accordingly. The frequency of authorships from SAARC countries was then calculated. In addition, the gender-wise distribution and contributions of each gender were determined.

## RESULTS

The characteristics of the top 20 ophthalmology journals included for year 2021 and 2022 are shown in Table-I. In 2022 rankings: Cornea, Journal of Refractive and Cataract Surgery, Journal of Refractive Surgery, and Ophthalmologica lost their spot in the top 20 and were superseded by Ophthalmology Glaucoma, Eye and Vision, Asia-Pacific Journal of Ophthalmology, and Ophthalmology and therapy. Therefore, the new entries were also considered. Most of the journals (n=13; 65%) in 2021 and (n=11; 55%) in 2022 were published from United States. Only the original articles were included from these years. As a result, the articles from the Journal of Progress in Retinal and Eye Research, Annual Review of Vision Science, Survey of Ophthalmology, and Current Opinion in Ophthalmology were excluded because these journals contained only review articles.

**Table-I:** Characteristics of top 20 ophthalmology journals based on SCImago journal rankings for year 2021 and 2022.

Year 2021					Year 2022		
Rank	Journal name	SJR	Country of origin	Rank	Journal name	SJR	Country of origin
1	Progress in Retinal and Eye Research	6.022	United Kingdom	1	Progress in Retinal and Eye Research	4.939	United Kingdom
2	Ophthalmology	4.412	United States	2	Ophthalmology	3.913	United States
3	Annual Review of Vision Science	3.038	United States	3	Annual Review of Vision Science	2.727	United States
4	JAMA Ophthalmology	2.311	United States	4	JAMA Ophthalmology	2.349	United States
5	American Journal of Ophthalmology	2.301	United States	5	Ocular Surface	1.983	United States
6	Survey of Ophthalmology	2.063	United States	6	American Journal of Ophthalmology	1.895	United States
7	Ophthalmology Retina	1.843	United States	7	British Journal of Ophthalmology	1.733	United Kingdom
8	British Journal of Ophthalmology	1.800	United Kingdom	8	Ophthalmology Retina	1.622	United States
9	Ocular Surface	1.685	United States	9	Survey of Ophthalmology	1.595	United States
10	Current Opinion in Ophthalmology	1.653	United States	10	Ophthalmology Glaucoma	1.280	United States
11	Retina	1.648	United States	11	Asia-Pacific journal of Ophthalmology	1.277	Netherlands
12	Eye	1.427	United Kingdom	12	Investigative Ophthalmology and Visual Science	1.259	United States
13	Investigative Ophthalmology and Visual Science	1.399	United States	13	Eye and Vision	1.223	United Kingdom
14	Journal of Cataract and Refractive Surgery	1.367	United States	14	Retina	1.206	United States
15	Cornea	1.336	United States	15	Eye	1.176	United Kingdom
16	Acta Ophthalmologica	1.314	United Kingdom	16	Current Opinion in Ophthalmology	1.132	United States
17	Graefe’s Archive for Clinical and Experimental Ophthalmology	1.305	Germany	17	Acta Ophthalmologica	1.128	United Kingdom
18	Journal of Refractive Surgery	1.298	United States	18	Eye and Brain	1.020	New Zealand
19	Eye and Brain	1.251	New Zealand	19	Ophthalmology and Therapy	1.020	United Kingdom
20	Ophthalmologica	1.245	Switzerland	20	Graefe’s Archive for Clinical and Experimental Ophthalmology	1.019	Germany

SJR=SCImago Journal Rank, JAMA=Journal of the American Medical Association.

**Fig.1:**
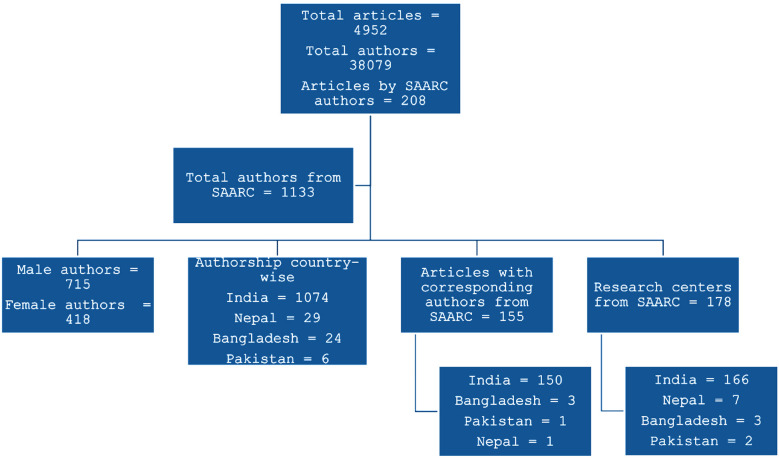
Details of authorship and contribution from SAARC region.

A total of 4952 ophthalmic original research articles published in 2021 and 2022 in top 20 journals were written by 38079 authors. There were 1133 (2.97%) authors from the SAARC region and 208 (4.2%) articles had authorship from the member countries. Male authors contributed more to SAARC region (n=715; 63.1%). Based on country-wise contribution, 1074 (94.8%) authors were from India, 29 (2.56%) authors from Nepal and 24 (2.12%) from Bangladesh. In 155 (3.13%) articles, the corresponding authors were from the SAARC region, among which 150 (96.78%) were from India. Out of 178 research centres involved from the SAARC region in 167 articles, 166 (93.26%) centres were in India. The details of authorship are shown in Fig.1. The country-wise contributions in top 20 ophthalmology journals based on SCImago rankings for year 2021 and 2022 is shown in Table-II.

**Table-II:** Country-wise contributions in top 20 ophthalmology journals based on SCImago rankings for year 2021 and 2022.

Year 2021					Year 2022				
Rank	Journal name	Authors from SAARC	Corresponding authors from SAARC	Research centers from SAARC	Rank	Journal name	Authors from SAARC	Corresponding authors from SAARC	Research centers from SAARC
1	Progress in Retinal and Eye Research	Excluded			1	Progress in Retinal and Eye Research	Excluded		
2	Ophthalmology	India=28 Nepal=8 Total=36	India=4	India:6 Nepal:2 Total=8	2	Ophthalmology	India =16 Nepal=7 Total=23	0	India=3 Nepal=1 Total=4
3	Annual Review of Vision Science	Excluded			3	Annual Review of Vision Science	Excluded		
4	JAMA Ophthalmology	0	0	0	4	JAMA Ophthalmology	India=25	India=2	India=4
5	American Journal of Ophthalmology	India=114	India=16	India=13	5	Ocular Surface	India=1	0	0
6	Survey of Ophthalmology	Excluded			6	American Journal of Ophthalmology	India=54	India=7	India=7
7	Ophthalmology Retina	India=13	India=1	India=3	7	British Journal of Ophthalmology	India=68	India=8	India= 8
8	British Journal of Ophthalmology	India=98 Bangladesh=7 Total=105	India=14 Bangladesh=1 Total=15	India=13 Bangladesh=1 Total=14	8	Ophthalmology Retina	India=22	India=1	India=7
9	Ocular Surface	India=11	India=1	India=1	9	Survey of Ophthalmology	Excluded		
10	Current Opinion in Ophthalmology	Excluded			10	Ophthalmology Glaucoma	India=36	India=6	India=6
11	Retina	India= 46	India=8	India=7	11	Asia-Pacific journal of Ophthalmology	Bangladesh=17	Bangladesh=2	Bangladesh=2
12	Eye	India=114 Pakistan=1 Total=115	India=16	India=15	12	Investigative Ophthalmology and Visual Science	India=9	India=2	India=2
13	Investigative Ophthalmology and Visual Science	India=47	India=7	India=7	13	Eye and Vision	India=1	0	India=1
14	Journal of Cataract and Refractive Surgery	India=23	India=7	India=7	14	Retina	India:46 Pakistan=3 Total=49	India=8 Pakistan=1 Total=9	India=8 Pakistan=1 Total=9
15	Cornea	India=17	India=3	India=4	15	Eye	India=76 Nepal=4 Total=80	India=15	India=16 Nepal=1 Total=17
16	Acta Ophthalmologica	India=8	India=1	India=1	16	Current Opinion in Ophthalmology	Excluded		
17	Graefe’s Archive for Clinical and Experimental Ophthalmology	India=102 Nepal=3 Pakistan=2 Total=107	India=13	India=13 Nepal=1 Pakistan=1 Total=15	17	Acta Ophthalmologica	India=4	India=1	India=1
18	Journal of Refractive Surgery	0	0	0	18	Eye and Brain	0	0	0
19	Eye and Brain	0	0	0	19	Ophthalmology and Therapy	India=29	India=1	India=5
20	Ophthalmologica	India=6	India=1	India=1	20	Graefe’s Archive for Clinical and Experimental Ophthalmology	India=60 Nepal=7 Total=67	India=7 Nepal=1 Total=7	India=7 Nepal=2 Total=9

JAMA=Journal of the American Medical Association.

**Fig.2:**
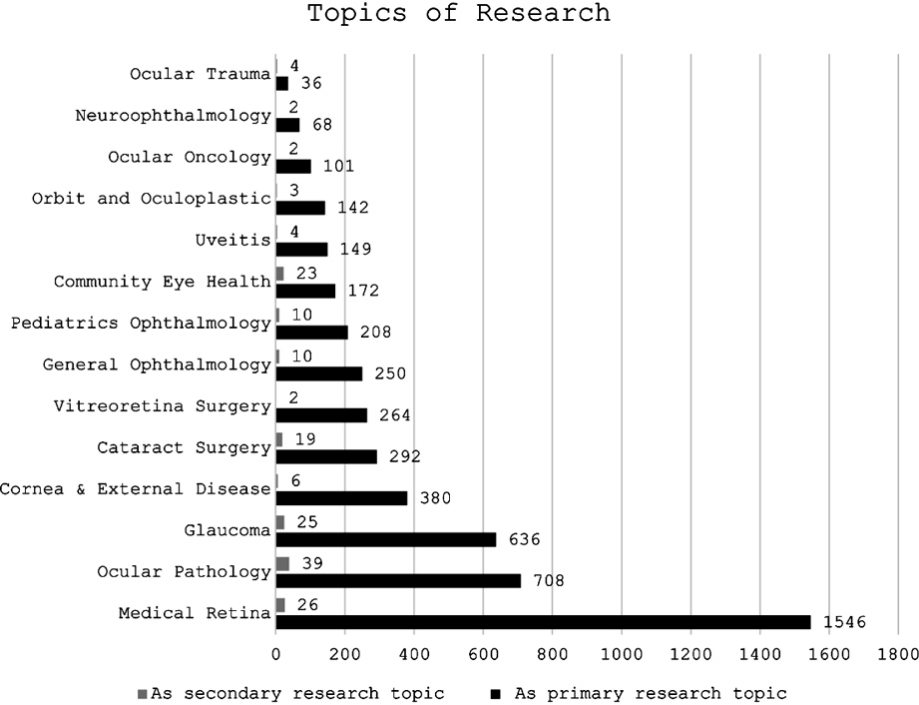
Topics of research interest (this includes contribution of all authors).

**Fig.3:**
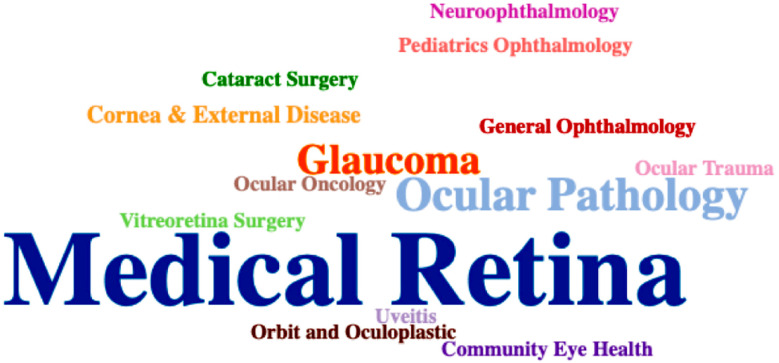
Word cloud representation of the topics of research.

The primary and secondary topics for research for all articles screened were also analysed and are shown in Fig.2, medical retina (n=1546; 31.22%) was the most common topic followed by ocular pathology (n=708; 14.3%) and glaucoma (n=636; 12.84%) as primary topics of research. Ocular pathology (n=39; 0.79%), medical retina (n=26; 0.52%), and glaucoma (n=25; 0.5%) were the leading secondary topics of research in these articles. Fig.3 highlights the overall representation of research topics as word cloud.

## DISCUSSION

Investigating the authors’ contributions to the top 20 ophthalmology journals for two years (2021 and 2022) from the SAARC region was the goal of this study. Although the proportion of SAARC authors relative to the global author population was 2.97%, they contributed to 4.2% of the original research articles published during these years. While there are no prior studies that are explicitly about the contributions of ophthalmic research from the SAARC region that we can compare with, we found a study on the state of research and development in the region. In four key scientific categories, Ahmad et al. revealed that 4.14% of articles in Nature Index journals came from the SAARC region.^5^ Our findings are in line with theirs, which indicates that the contribution to ophthalmic research is equivalent to that of other scientific fields. According to a study by Heng Wong and colleagues, they did not find citations from the SAARC region among the top 100 most-cited ophthalmology articles.^11^ Likewise, Fonteno and Liu found that the top 10 contributors to publications of original research, such as clinical trials, did not include any representation from the SAARC region in their study based on a PubMed search.^12^ This low level of contribution from the SAARC region to ophthalmic research could be attributed to several reasons, including policy and governance, education and training, access to technology and innovation, inadequate research infrastructure, resource constraints, and collaboration and networking.

According to a previous report, the tendency of collaborative research is lower in the SAARC region than outside of it (2.2% versus 20%), and this may be an important contributing factor.^5^ The under representation of SAARC region work on the international scene could be due to the absence of high impact factor journals from the region as highlighted by our results (Table-I). Prior research has indicated that authors from the United States and the United Kingdom who publish in top journals tend to cite their own journals more frequently than any other country.^13^ Additionally, American reviewers significantly favour publications from the United States.^14^ These could be the explanations for the high number of citations to US and UK authors.

India contributed more than any other country in the SAARC region to ophthalmic research: 94.8% of authors, 96.78% of corresponding authors, and 93.26% of research centres. In the SCImago country rankings for ophthalmology in 2021 and 2022, India was ranked third. In 2022, India published a total of 2472 documents in the field of ophthalmology, up from 2262 in 2021.^15^,^16^ In the top 100 most-cited publications from Asia, India dominated the SAARC region, accounting for 8-10% of the citations.^1^ India’s substantial contribution could be attributed to many factors, including its large population, prevalence of disease, distinguished academic and medical institutions, global collaboration, private sector engagement, and economic prosperity.

Evidence highlights a strong positive association between the nation’s economic growth and its scientific output or publications.^17^ India has shown significant improvement in its gross domestic product (GDP) in the last few years.^18^ Another important indicator is Gross Expenditure on Research and Development (GERD) where India is excelling in the SAARC region.^19^ The recent development of the BRICS (Brazil, Russia, India, China and South Africa) countries-of which India is one of the founding members-might be another significant explanation. Menon et al compared the India’s scientific research presentations in pharmacoeconomic and health outcomes research to those of the BRICS and SAARC countries.^20^ They concluded that the BRICS countries have seen a noticeable increase in pharmacoeconomic and health outcomes research over the last ten years. Research in this area shows that the BRICS nations are developing on a forward-leaning path. Overall, compared to other SAARC nations, India produces more ophthalmology research, which can be because of its robust research infrastructure, rapid economic growth, and extensive networks of collaboration in the field. India’s experience must be emulated by other SAARC nations who also need to boost their research output.

The other SAARC nations that contributed to ophthalmic research in these journals in 2021 and 2022 were Bangladesh, Nepal, and Pakistan. In the SCImago country rankings for ophthalmology, Bangladesh, Nepal, and Pakistan were ranked 80^th^, 52^nd^, and 36^th^ in 2021 and 61^st^, 52^nd^, and 35^th^ in 2022, respectively. In 2021, Bangladesh, Nepal, and Pakistan contributed 10, 33, and 85 papers; in 2022, those numbers increased to 20, 37, and 92 documents, respectively.^15^,^16^ While these countries have shown improvement in contribution to ophthalmic research they need a lot of hard work. Pakistan contributed to the emerging topics of research from Asia.^1^ The low output of these countries may be due to the same challenges faced by ophthalmologists from Sub-Sahara countries like limited funding, research training, access to quality journals, and research incentives.^21^ A comparable study of Arabs’ contributions to ophthalmic research between 1900 and 2012 was carried out by Arab researchers. They reported that during the study period, 2035 original articles were retrieved from the 21 Arab nations.^22^ The authors suggested that Arabs working in the field of ophthalmology should connect and cooperate on research with other highly developed nations. In our study, most research topics focused on medical retina, glaucoma, and ocular pathology. Prior research has also demonstrated that glaucoma and retina consistently rank among the most popular topics for study.^1^,^4^,^23^,^24^ The reasons may be that there are constantly evolving therapeutic and diagnostic procedures in these two fields, as well as the presence of the retina, glaucoma, and ocular surface journals, which ranked higher than other subspecialties in the top 20, and may have influenced these findings.

To the best of our knowledge, no other study has investigated the contribution of ophthalmologists from the SAARC region to ophthalmic research. One advantage of our study is that, by examining the contribution of ophthalmologists from the SAARC to research, our work pioneers a new territory. This unique perspective broadens our understanding of the field and enhances the existing literature. Our study offers a thorough review of the research contributions of ophthalmologists in the SAARC region. Our research may help shape policies intended to improve ophthalmology’s research capacity. Our study is likely to facilitate future collaborations between researchers and organizations in the SAARC region and rest of the world.

### Limitations:

Our study has certain limitations, for example, our data may have been constrained because we focused on the top 20 ophthalmology journals as per SCImago journal rankings. The authors from the SAARC region may have contributed to top non-ophthalmology journals but due to our stringent inclusion criteria, they have not been counted. We only included the original research articles that excluded the other types of research contributions like editorials, reviews, meta-analyses, and case reports so the SAARC region’s authors’ contribution may be underrepresented. Research articles from the SAARC area may be published in languages other than English which could make it difficult to access and include them in our analysis. Language barriers could cause our findings to be skewed and result in insufficient coverage of pertinent research.

SAARC ophthalmologists may encounter difficulties in publishing their research in top international journals because of factors like low institutional support, language limitations, and resource accessibility. Publication bias may have an impact on how research contributions are represented in our analysis, which could lead to underestimation of the actual volume of ophthalmic research conducted in the area. The ophthalmologists of SAARC may focus on a variety of subspecialties, from public health and epidemiology to clinical practice. The scope and breadth of our study may have been affected by researchers’ varying research interests, which makes it difficult to reach firm conclusions on the total amount of research contributed.

## CONCLUSION

Despite hosting quarter of the whole world’s population, the SAARC region makes a small contribution to ophthalmic research in top 20 ophthalmology journals. Research contribution seems to be proportional to region’s economic contribution to that of world’s economy with India representing the maximum contribution to the original research published.

### Future Recommended:

It is recommended that a valuable global perspective should be obtained by comparing the scientific contributions of ophthalmologists from the SAARC region with those from other regions. This comparative analysis might contribute to more extensive debates in the field and identify the opportunities and challenges experienced by ophthalmologists in the SAARC area. There is not a single ophthalmology journal in top 20 from SAARC region which South Asian Academy of Ophthalmology must consider an important area to work on. By addressing this challenge, it will open doors for the researchers from the region to share their work on global platform. Moreover, to address the obstacles that the researchers face; governments, universities, funding organizations, and international organizations from the SAARC region would need to work together to invest in research infrastructure, offer chances for training and education, encourage collaboration, and put supportive policies in place. This will help advance ophthalmic research in the SAARC region.

### Authors’ Contribution:

**YJM:** Concepts, Data acquisition, Manuscript drafting, Manuscript editing,

**RK & HW:** Design, Data acquisition Data analysis & interpretation, Manuscript drafting, Manuscript editing, Final approval and agreed to be accountable.

**SF & RZ:** Concepts, Design, Manuscript drafting, Manuscript editing, Final approval and agreed to be accountable.

**YJW, RK, HW, SF and RZ:** Final approval and agreed to be accountable.

## References

[1] Koh BMQR, Banu R, Sabanayagam C (2020). The 100 Most Cited Articles in Ophthalmology in Asia. Asia-Pacific J Ophthalmol.

[2] Bro T (2022). Worldwide ophthalmological research production 2000–2020, with special focus on the Nordic contribution. Acta Ophthalmol.

[3] Wikimedia Foundation I (2004). South Asian Association for Regional Cooperation - Wikipedia [Internet].

[4] Fu Y, Mao Y, Jiang S, Luo S, Chen X, Xiao W (2023). A bibliometric analysis of systematic reviews and meta-analyses in ophthalmology. Front Med (Lausanne).

[5] Ahmad A, Ashraf S, Abbas Z (2016). Current Status of Research and Development in the SAARC Region. Sci Technol Dev.

[6] Akhtar S, Shah SWA, Rafiq M, Khan A (2016). Research design and statistical methods in Pakistan Journal of Medical Sciences. Pak J Med Sci.

[7] Baladi ZH, Umedani LV (2017). Pakistan Journal Of Medical Sciences:A bibliometric assessment 2001-2010. Pak J Med Sci.

[8] Journal Rankings on Ophthalmology [Internet] (2022). Scimago J Country Rank.

[9] Journal Rankings on Ophthalmology [Internet] (2022). Scimago J Country Rank.

[10] Chien JL, Wu BP, Nayer Z, Grits D, Rodriguez G, Gu A (2020). Trends in authorship of original scientific articles in journal of glaucoma:An analysis of 25 years since the initiation of the journal. J Glaucoma.

[11] Heng Wong MY, Tan NYQ, Sabanayagam C (2019). Time trends, disease patterns and gender imbalance in the top 100 most cited articles in ophthalmology. Br J Ophthalmol.

[12] Fontelo P, Liu F (2018). A review of recent publication trends from top publishing countries. Syst Rev.

[13] Campbell FM (1990). National bias:A comparison of citation practices by health professionals. Bull Med Libr Assoc.

[14] Link AM (1998). US and non-US submissions. An analysis of reviewer bias. JAMA.

[15] Scimago Journal and Country Rank SJR - International Science Ranking [Internet].

[16] Scimago Journal and Country Rank SJR - International Science Ranking [Internet].

[17] Ahmad A, Ashraf S, Raheem A, Yasmeen A (2014). Science, Scientists and Economic Growth. Sci Technol Dev.

[18] Banco Mundial. GDP (current US$) |Data [Internet] (2022). The World Bank.

[19] Schulz CB, Kennedy A, Rymer BC (2016). Trends in ophthalmology journals:A five-year bibliometric analysis (2009-2013). Int J Ophthalmol.

[20] Menon S, Nayak H, Ligade V (2019). Scientific research presentations in pharmacoeconomics and health outcomes research:Status of India compared to SAARC and BRICS countries. J Appl Pharm Sci.

[21] Dhalla KA, Guirguis M (2018). Barriers and incentives for conducting research amongst the ophthalmologists in Sub-Sahara Africa. PLoS One.

[22] Sweileh WM, Al-Jabi SW, Shanti YI, Sawalha AF, Zyoud SH (2015). Contribution of Arab researchers to ophthalmology:a bibliometric and comparative analysis. Springerplus.

[23] Koh BMQR, Banu R, Nusinovici S, Sabanayagam C (2021). 100 most-cited articles on diabetic retinopathy. Br J Ophthalmol.

[24] Boudry C, Denion E, Mortemousque B, Mouriaux F (2016). Trends and topics in eye disease research in PubMed from 2010 to 2014. Peer J.

